# Microbeam Irradiation as a Simultaneously Integrated Boost in a Conventional Whole-Brain Radiotherapy Protocol

**DOI:** 10.3390/ijms23158319

**Published:** 2022-07-28

**Authors:** Felix Jaekel, Elke Bräuer-Krisch, Stefan Bartzsch, Jean Laissue, Hans Blattmann, Marten Scholz, Julia Soloviova, Guido Hildebrandt, Elisabeth Schültke

**Affiliations:** 1 Department of Radiooncology, Rostock University Medical Center, 18059 Rostock, Germany; felix@m20a.de (F.J.); martenlscholz@hotmail.de (M.S.); julia.soloviova@uniklinik-leipzig.de (J.S.); guido.hildebrandt@uni-rostock.de (G.H.); 2 Biomedical Beamline ID 17, European Synchrotron Radiation Facility (ESRF), 38043 Grenoble, France; krisch@esrf.fr; 3 Department of Radiooncology, Technical University of Munich, 81675 Munich, Germany; stefan.bartzsch@tum.de; 4 Institute for Radiation Medicine, Helmholtz Center Munich, 85764 Munich, Germany; 5 Institute of Anatomy, University of Bern, 3012 Bern, Switzerland; jean-albert.laissue@pathology.unibe.ch; 6 Niederwiesstr 13C, 5417 Untersiggenthal, Switzerland; hans.blattmann@bluewin.ch; 7 Department of Paediatric Surgery, Leipzig University Medical Centre, 04103 Leipzig, Germany

**Keywords:** microbeam radiotherapy (MRT), simultaneously integrated boost (SIB), brain tissue tolerance, F98 glioma cells

## Abstract

Microbeam radiotherapy (MRT), an experimental high-dose rate concept with spatial fractionation at the micrometre range, has shown a high therapeutic potential as well as good preservation of normal tissue function in pre-clinical studies. We investigated the suitability of MRT as a simultaneously integrated boost (SIB) in conventional whole-brain irradiation (WBRT). A 174 Gy MRT SIB was administered with an array of quasi-parallel, 50 µm wide microbeams spaced at a centre-to-centre distance of 400 µm either on the first or last day of a 5 × 4 Gy radiotherapy schedule in healthy adult C57 BL/6J mice and in F98 glioma cell cultures. The animals were observed for signs of intracranial pressure and focal neurologic signs. Colony counts were conducted in F98 glioma cell cultures. No signs of acute adverse effects were observed in any of the irradiated animals within 3 days after the last irradiation fraction. The tumoricidal effect on F98 cell in vitro was higher when the MRT boost was delivered on the first day of the irradiation course, as opposed to the last day. Therefore, the MRT SIB should be integrated into a clinical radiotherapy schedule as early as possible.

## 1. Introduction

The currently available options in the treatment of malignant brain tumors such as anaplastic astrocytoma or glioblastoma multiforme (WHO Grade III and Grade IV, respectively) are limited and the long-term prognosis is poor. This is especially true for multifocal glioblastoma, where the progression-free survival (PFS, time to first recurrence) has been reported as 6.6 months [1]. Multifocality can be explained by tumour cell migration along the so-called Scherer’s structures [2,3,4]. Therefore, beyond local measures such as the surgical removal of visible tumour structures and radiotherapy of the tumour bed including the edema zone, a successful therapeutic approach should include a treatment component which eliminates tumour cells that have migrated to locations distant from the primary tumour site.

Historically, a statistically significant increase in survival time has been achieved with the introduction of a simultaneous radiochemotherapy protocol including temozolamide [5]. However, patients become refractory to the chemotherapeutic compound. The reported overall median survival of patients with glioblastoma multiforme is little more than one year [6,7]. Median overall survival appears to be slightly better for female (16.9 months) as compared to male patients (15.7 months) and males seem to be more frequently affected than females [8]. While the more recent introduction of the CeTeG radiochemotherapy protocol might add a few more months to survival for patients with a methylated MGMT promotor [9], the overall prognosis is still far from satisfactory.

Since the introduction of simultaneous radiochemotherapy with temozolomide, an up to 21.5% increased number of patients is seen with tumor recurrence, with a positive correlation to supraventricular tumor location and methylated MGMT status [10,11]. Therapeutic options are a second surgical intervention and re-radiotherapy. Considering the migration patterns of malignant brain tumour cells, whole-brain radiotherapy (WBRT) would appear to be a logical choice. However, WBRT with current clinically established radiotherapy techniques is not considered an option for treatment of the primary tumour because the high X-ray doses required to control the brain tumor cells would cause an extremely high risk of cognitive dysfunction, especially impaired memory function [12,13].

Microbeam radiotherapy (MRT), a new concept of high-dose rate radiotherapy with a spatial dose fractionation at the micrometer range, might provide a good compromise between a satisfactory tumor control in the entire brain and functional preservation. The therapeutic efficacy of MRT has been demonstrated in several small animal models of malignant brain tumor [14,15,16] while the normal brain tissue tolerance appears to be remarkably high [17,18]. Memory function appears to be well preserved [19].

It has already been shown that MRT that is focused on the macroscopic tumour, as a single fraction or included in a conventional radiotherapy schedule, can control the tumour much better than conventional radiotherapy alone [20,21]. In a model of young adult Fisher rats bearing a highly malignant brain tumour generated from implanted F98 glioma cells, three fractions of conventionally fractionated broad beam irradiation administered by an X-ray generator (orthovoltage range) were followed by two fractions of synchrotron-based MRT [20]. The authors concluded that MRT, delivered as a sequential boost after conventionally fractionated broad beam irradiation at the orthovoltage range, is feasible and more efficient than conventional radiotherapy alone.

We have now, for the first time, explored the concept of WBRT combined with MRT. Our data, obtained in a small animal model, support the idea that microbeam WBRT is feasible and that it can be integrated into a conventional radiotherapy schedule as simultaneously integrated boost without acute adverse effects.

## 2. Results

### 2.1. Survival, Behaviour and Normal Tissue Function

**Figure 1:**
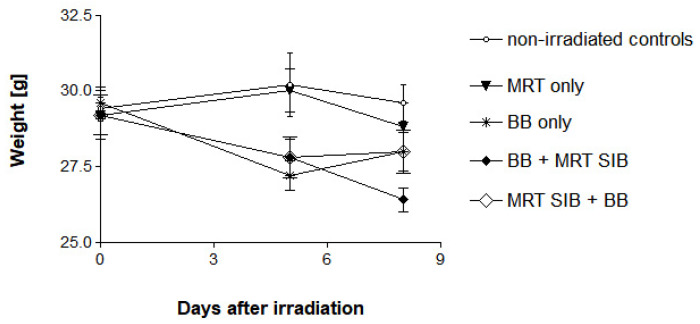
Weight development within 8 days after the first irradiation fraction. The most pronounced weight loss was seen in the animals receiving the MRT SIB at the end of a broad beam irradiation schedule. BB: broad beam radiotherapy, MRT: microbeam radiotherapy.

In this experiment, no deaths occurred in any of the experimental groups. No signs of increased intracranial pressure, such as circling, expressions of pain or apathy, generalised or focal epileptic seizures occurred in any of the animals. There appeared to be slight weariness and reduction in appetite in all animals which received a five-day irradiation course, associated with slight weight loss (Figure 1). The weight loss was most pronounced in the animals which received an MRT SIB at the end of the irradiation schedule (average of 11.7% on the eighth day after the first irradiation, compared to the initial weight). In the animals who received the MRT SIB at the beginning of the irradiation schedule, the weight loss was approximately equal to that seen in the five-day broad beam schedule (average of 10.0 and 9.1%, respectively, on the eighth day after the first irradiation, compared to the initial weight).

The observation of weariness and inappetence is not dissimilar to the clinical observations made in human patients who receive whole-brain radiotherapy. Furthermore, the need for increased doses of anaesthetics to achieve anaesthesia sufficiently deep to guarantee immobility during the irradiation was noticed in approximately two thirds of the animals, starting with the fourth irradiation fraction, regardless of whether the MRT SIB was the first or the last fraction of the irradiation schedule.

**Figure 2:**
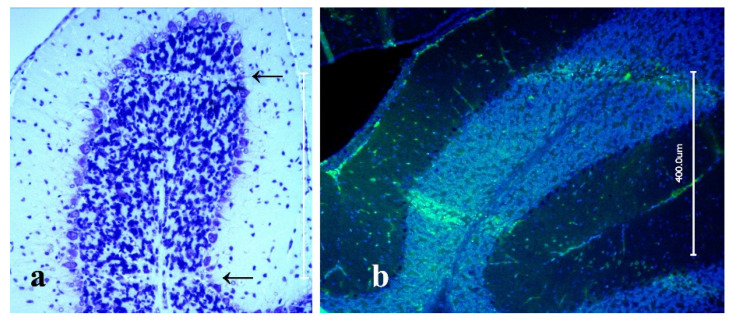
Section through the cerebellum of an adult rat after MRT SIB with a microbeam peak dose of 174 Gy, followed by four fractions of 4 Gy broad beam radiotherapy (BB) on four subsequent days, at 24 h after the last irradiation fraction. Nissl stain with reduced number of cells in the microbeam paths (arrows; vertical scale bar approximates 400 µm) (**a**) and immunostaining with DAPI counterstain (blue) and gamma H2AX antibody (green), highlighting DNA double strand breaks (**b**). DNA double strand breaks are especially numerous in the paths of the microbeams. The bright linear paths of the microbeams can be well-visualised in the cerebellum because of the local density of cell bodies.

Nissl staining showed thin lines of cell degradation and apoptosis corresponding to the microbeam pattern (Figure 2a). However, the general structure of the tissue remained intact. It has been shown previously that, despite microscopic morphologic defects, tissue function is preserved [18,22]. Applying gamma-H2AX immunostain, we could see a geometrical correlation of DNA double strand breaks with the irradiation pattern, including both a diffuse staining of lower intensity across the entire brain as consequence of the whole-brain irradiation and the typical intense staining in the paths of the microbeams (Figure 2b).

### 2.2. In-Vitro Study with Clonogenic Assays Using F98 Glioma Cells

**Figure 3:**
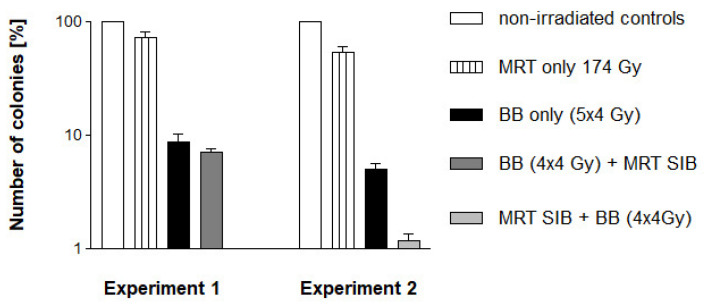
Colony counts of F98 glioma cells submitted to the same irradiation schedules used for the in vivo study. The number of colonies grown as a percentage is shown, compared to the number of cells originally seeded in each flask. MRT: Microbeam radiotherapy, BB: broad beam generated with the X-ray generator.

A single session of MRT reduced the clonogenic potential of the F98 cells to 61.6% (Experiment 1) and 50.3% (Experiment 2), while the five fractions of conventional broad beam radiotherapy managed to reduce colony counts to 8.7% (Experiment 1) and 5.0% (Experiment 2). When the MRT SIB was administered at the end of the conventional radiotherapy schedule (four fractions BB + MRT SIB), the number of colonies was reduced to 7.0%. There was a trend to improved cell destruction but there was no statistically significant difference in the number of viable colonies, compared to the five fractions of conventional broad beam radiotherapy (BB alone). However, when the MRT SIB was administered at the beginning of the radiotherapy schedule, the number of viable colonies formed was only approx. 1%, which was statistically significant (*p* = 0.0004) (Figure 3).

Thus, the administration of the MRT SIB at the beginning of the conventional radiotherapy schedule resulted in a reduction of viable tumour cell colonies by two orders of magnitude, while the administration of five fractions of broad beam irradiation or the administration of the MRT SIB at the end of the conventional radiotherapy schedule resulted in five to eight times more viable tumour cell colonies left.

Based on the observations that, in vivo, no acute adverse effects occurred after administration of the MRT SIB either at the beginning or at the end of the conventional radiotherapy schedule and that the reduction of viable tumour cell mass, in vitro, was significantly lower after administration of the MRT SIB at the beginning of the conventional radiotherapy schedule, we conclude that the administration of the MRT SIB at the beginning of the radiotherapy schedule is recommendable.

## 3. Discussion

The concept of WBRT is well-established for the treatment of patients with multiple brain metastases; often supporting and successfully decreasing or completely ablating neurologic symptoms. Conversely, WBRT has not yet found a place in the treatment of patients with high grade glioma because the toxicity to the normal brain tissue is a limiting factor.

The introduction of simultaneous radiochemotherapy based on the concept of Stupp, has increased the overall survival of patients with high-grade, malignant brain tumors by several months. The number of patients with tumor recurrence has increased by more than 20%, especially in the subgroup of patients with a methylated MGMT promotor, the subgroup with the highest survival benefit [5,7]. Based on the knowledge of tumor cell migration in high-grade gliomas, the incidence of tumour recurrence can be expected to increase further with an increase in overall survival times. Therapy failure can occur due to local or distant tumor recurrence and is likely to be due to tumor cells surviving the first radiochemotherapy. Therefore, patients would benefit from a therapeutic component which eliminates all the tumor cells that have migrated out of the primary tumor site and thus, out of the irradiation field, covering the primary tumor and its surrounding edema zone.

In this preclinical study we have shown for the first time that no acute adverse effects occurred during or within the first days after the end of the irradiation schedule where a single fraction of monoplanar uni-directional microbeam WBRT (MRT SIB) was integrated into a clinical broad beam WBRT schedule. There were no fatalities, no motoric deficits and no signs of intracranial pressure, such as decreased activity, loss of appetite, epileptic seizures or circling, regardless of whether the MRT SIB was introduced in the beginning or the end of the irradiation schedule.

Other than in clinically established radiotherapy techniques, where the gantry is moved around the patient, the synchrotron beam used for microbeam irradiation at the ESRF is fixed in position and the patient needs to be moved through the beam to irradiate the entire target volume. Therefore, contrary to WBRT with broad beam techniques, where irradiation is delivered from two coplanar ports positioned at an angle of 180°, MRT was administered from one port and in one direction only, because patient re-positioning for MRT with the accuracy that would be required to match the paths of the microbeams of the first MRT fraction exactly is impossible with the currently available techniques. However, it has already been shown that two boost fractions of monoplanar uni-directional MRT focused on the tumour site, administered in different planes after a conventional radiotherapy schedule for focal tumours, increases survival significantly [20]. On the basis of our observations in the present study, we suggest delivering the MRT SIB to the side of the hemisphere in which the tumour is located.

To further increase the recurrence-free survival interval after re-RT, one could cross the WBRT microbeams coplanar at an angle of 90° to include the location of the macroscopic tumor recurrence only. This would result in microbeam peak doses of 348 Gy, a dose that did not cause serious adverse effects and impacted only minimally on new memory formation in earlier studies [18,19]. The crossed beams, covering part of the normal brain tissue, would result in a nominal valley dose of 8 Gy, which should be below the accepted threshold for cerebral necrosis. Tumor cells respond to MRT differently than normal brain cells. Malignant tumor cells, with their much higher proliferation rate, compared to normal brain tissue, have a much higher potential to incur radiation-induced damage. Furthermore, it has been reported that the tumour vasculature is much more sensitive and therefore liable to get damaged by microbeam irradiation, rather than mature, normal blood vessels [17]. It is important to test these characteristics of MRT in veterinary patients, since some breeds of dogs suffer from brain tumors very similar to those seen in human patients, in both histological characteristics and the course of the disease [23]. Based on data obtained in small animal models, MRT may also help to control malignant melanoma [24] and lung cancer [25].

The 5 × 4 Gy WBRT schedule is typically used to treat patients with multiple intracerebral metastases in an advanced stage of the disease. We have chosen this clinical irradiation schedule for logistic reasons and also to demonstrate that the WBRT MRT SIB concept would be a suitable alternative in the treatment of patients with multiple brain metastases. For the treatment of recurrent glioblastoma, a 13 × 3 Gy schedule with a focal EQD2 of 48.75 Gy and a biologically equivalent dose (BED) of 97.5 Gy is more common. The EQD2 in our study was 30 Gy and the BED was 60 Gy in the entire brain, based on the valley dose alone. Based on the valley dose again, one additional fraction of 4 Gy broad beam irradiation would bring the EQD2 in the entire brain up to 36 Gy and the BED up to 72 Gy. However, this may not be necessary if an MRT SIB is administered. Differential effects between normal and tumor tissue have been widely reported in the literature of high dose rate synchrotron MRT. The effects of ultra-high dose rates (also called FLASH dose rates) have been shown to significantly reduce normal tissue toxicity while being as efficient as conventional dose rate radiotherapy with regard to tumour destruction. Furthermore, the impressive therapeutic ratio combined with the exceptional resistance of normal tissues to MRT may at least partially be attributed to its unique dose–volume effect [26].

The results of the in vitro study in F98 glioma cells, accompanying our small animal study at irradiation conditions similar to those used for the in vivo study, show that one single fraction of monoplanar MRT significantly decreased the clonogenic potential of the tumour cells when integrated in a conventional broad beam radiotherapy schedule of five subsequent irradiation fractions, compared to either non-irradiated controls or cells irradiated with only one single fraction of MRT. We have observed that the smallest number of viable tumor cell colonies was seen at the end point of the observation period when the MRT SIB was administered at the beginning, rather than at the end of the irradiation schedule.

These results suggest that, to prevent multifocal tumor development, the elimination of tumor cells outside of the primary irradiation target should be approached early in the treatment process, to minimise the risk of clonogenic tumor growth outside of the irradiation field covering the primary tumor site.

A further important argument for the integration of the MRT SIB at the beginning rather than the end of the broad beam radiotherapy schedule: broad beam irradiation will predictably induce radiogenic damage to the tumor-supplying vasculature. This could significantly increase the risk of potentially fatal edema development or hemorrhage.

Once MRT has technically matured to the stage where it can be safely administered to human patients, it may become an important therapeutic component in the therapy of patients with high-grade, malignant brain tumors. A combination of MRT and conventional broad beam irradiation can increase the potential to deplete tumor cells and decrease the clonogenic potential, ensure a higher radiation tolerance in organs of risk, and thus improve patient safety. For recurrent tumors, a WBRT MRT SIB integrated into a conventional radiotherapy schedule might reliably eliminate tumor cells which have survived the first radiochemotherapy.

## 4. Materials and Methods

The experiments were conducted at the biomedical beamline ID17 of the European Synchrotron Radiation Facility (ESRF) in Grenoble, France (permit number 14ethax210 of the ESRF Ethics Committee, ETHAX 113, authorisation 28 May 2015).

### 4.1. Animal Model

Forty adult C57 BL/6J mice were used for this study, distributed into four experimental groups (n = 10/group):

Group 1 received five single fractions of 4 Gy WBRT on five subsequent days.

Group 2 and Group 3 received four fractions of 4 Gy WBRT on four subsequent days. In addition, the animals in Group 2 received on the fifth day one single fraction of WBRT MRT with a valley dose of 4 Gy while the animals in Group 3 received one single fraction of WBRT MRT with a valley dose of 4 Gy on the first day of the irradiation schedule. In other words, all animals of Groups 1–3 received 5 × 4 Gy delivered to the entire brain. On top of this (as a simultaneous boost), the animals in Group 2 and Group 3 received a microbeam peak dose of 174 Gy, which was calculated based on the target valley dose of 4 Gy. The beam geometry was recorded on self-developing radiosensitive Gafchromic™ film. The concept of the simultaneously integrated boost is frequently used in clinical radiotherapy, to increase biological efficacy and to shorten the overall treatment time in the interest of the patient, increasing the patient’s quality of life. The animals in Group 4 served as non-irradiated controls.

### 4.2. Irradiation Sources

Broad beam irradiation with an X-Ray generator (BB): broad beam radiotherapy was delivered using a conventional X-ray Generator (Philips) with a 0.2 mm copper filter at an Energy of 200 keV. The dose was measured in a water phantom at 1 cm depth.

Synchrotron source: At ID 17, the synchrotron-generated photon beam was modified by a wiggler set to its minimum gap (24.8 mm) in irradiation mode, to benefit from the maximum available photon flux, and passed through a set of Cu and Al filters. The spectrum used for MRT at this beamline is typically 50–350 keV, with a maximum intensity at approx. 105 keV [27]. The study was conducted in a lead-shielded hutch enclosure of the Insertion Device (ID) 17 beamline at the ESRF [28].

An array of quasi-parallel microbeams with an individual beam width of 50 µm spaced at a centre-to-centre distance of 400 µm was generated by inserting a fixed-space tungsten multislit collimator into the incident beam [29]. Previous experiments in small animal models with different beam configurations have shown those parameters to be a good compromise between therapeutic efficacy and the preservation of normal tissue function [30]. At the irradiation target, the microbeam array produces an inhomogeneous dose distribution, characterised by a repetitive pattern of high-dose (peak dose) zones and low-dose (valley dose) zones. A high peak-to-valley dose ratio (PVDR) is essential for good normal tissue preservation. A fast dose deposition is required to preserve a steep dose decrease at the microbeam edges and limit dose blurring at the beam edges through physiologic movement like heartbeat or breathing. The dose rate was measured by a semiflex ion chamber (PTW, Freiburg, Germany), scanning vertically through the beam at 100 mm/s, a 2 × 2 cm field at 2 cm depth in solid water. At machine storage ring currents between 152 and 198 mA, dose rates of approximately 70 Gy/s/mA were achieved at the irradiation position. Thus, the peak doses required in this study were delivered within a few seconds. Currently, this irradiation technique is only available at high energy synchrotron sources.

The rats were positioned prone, on top of a 3-axis Kappa-type goniometer (Huber, Rimsting, Germany). Three prosilica cameras (Allied Vision Technologies GmbH, Stadtroda, Germany) allowed a reproducible positioning of each animal. The microbeam irradiation of the entire skull was performed by a vertical translation of the rat through the beam. A fast shutter system [31], positioned upstream from the multislit collimator and synchronised with the vertical translation of the goniometer, allowed for the precise selection of the irradiation field and the pre-calculated speed, taking into account the decreasing current in the machine, thus precisely ensuring the delivery of the intended dose.

### 4.3. Dose Calculation and Modelling

**Figure 4:**
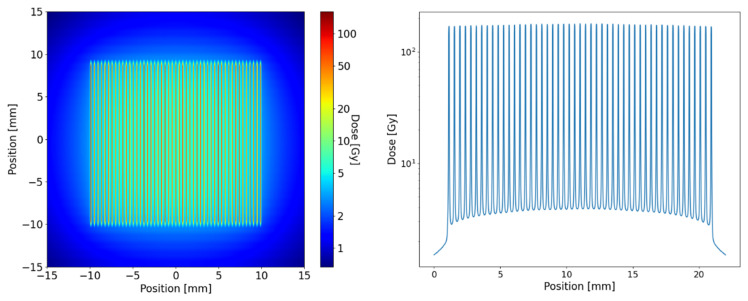
Results from the Monte Carlo simulation for irradiation with an MRT peak dose of 174 Gy at 1 mm depth in water (cell layer of in vitro exposures).

Calculations for the dose distribution inside the tissue (equivalent to 1 cm depth in water) were provided using Monte Carlo simulations in the toolkit GEANT4 version 10.4.2. All simulations used the Livermore low energy physics libraries, and range cut-offs for electrons and photons were set to 1 µm. Simulations were performed in its semi-adjoint form [32] and the source model was adapted from [33]. The field size was 20 × 20 mm² for the in vitro experiment and 8.5 × 18 mm² for the mouse experiment. The microbeams hit the water phantom of 20 µm width and energy was scored at a mesh size of 1 × 1 × 0.005 mm with the highest resolution perpendicular to the microbeam planes. A total number of 10^9^ photons were simulated following the ESRF preclinical spectrum [27] and collimator leakage with a harder X-ray spectrum was taken into account [33]. Figure 4 shows the simulated microbeam dose profile for the in vitro exposures.

In the centre of the radiation field the maximum peak dose in the in vitro and in vivo experiment was 174 Gy. The maximum valley dose was 3.5 and 4.4 Gy in the in vitro and in vivo exposures, respectively. However, the valley doses varied substantially across the radiation field and were around 30% lower at the field edges.

To compare the highly inhomogeneous dose distribution of the X-ray microbeams with BB exposures, the concept of equivalent uniform dose (EUD) was used as originally defined by Niemierko [34]. The EUD is the homogeneous dose that leads to the same cellular survival as an inhomogeneous dose distribution, assuming that cells react independent of each other to the local dose they receive according to the linear quadratic model (LQM). The LQM parameters *α* and *β* were assumed to be 0.1 Gy^−1^ and 0.05 Gy^−2^. Hence, the EUD was retrieved by equating the homogeneous and inhomogeneous survival using

$$\bar{S}=exp\left(-\alpha \cdot EUD-\beta \cdot {\mathrm{EUD}}^{2}\right)=\frac{1}{V}\int_{V}d^{3}\overset{\to }{r}\;e^{-\alpha \cdot D\left(\overset{\to }{r}\right)-G\cdot \beta \cdot D^{2}\left(\overset{\to }{r}\right)}.$$

For the mice treatments the EUD at a 1 cm depth (approximately the position of the brain) was 4.7 Gy and in the in vitro exposures were 6.0 Gy. The EQD^2^ of the entire course of the fractionated treatment for the BB only (5 × 4 Gy), the in vivo MRT SIB + BB (4 × 4 Gy + 6 Gy) and in vitro MRT SIB + BB (4 × 4 Gy + 4.7 Gy) was 30, 32 and 36 Gy, respectively.

### 4.4. In Vivo Model

Adult C57 BL/6J mice (Charles River, France) weighing between 280 and 320 g were used for the normal tissue response study. The animals were housed and cared for in a temperature-regulated animal facility exposed to a 12-h light/dark cycle.

Irradiation was conducted under general anaesthesia, induced by inhalation of 1.5–2% Isoflurane in compressed air and upheld by an intraperitoneal injection of a Ketamine and Xylazine cocktail (Ketamine 1 mg/10 g, Xylazine 0.1 mg/10 g). The anaesthetised mice were placed on a special positioning device in prone position, with the front teeth hooked into a holding device to assure a reproducible position.

Irradiation on the conventional X-ray generator for broad beam irradiation was conducted from above, in a dorsal-to-ventral direction. MRT was conducted in right-to-left lateral direction.

Prior to MRT, a 2D X-ray image was obtained, after which the target position was corrected, if necessary, to target the brain only and spare other tissue as much as possible. The animals were sacrificed at 24 and 72 h after the last irradiation. The brains were carefully extracted from the skull, fixed in 10% phosphate-buffered formalin for 24 h and then stored in 1× PBS for later processing.

### 4.5. In Vitro Model

In order to assess whether the irradiation schedule tested in our experiment would be tumouricidal in glioma cells, we conducted an in vitro study using the commercially available F98 glioma cell line (CRL-2397, ATCC, Manassas, VA, USA, rodent origin). Due to characteristics such as a high proliferation rate and invasive growth into normal brain structures, F98 glioma cells are frequently used to simulate the malignant human brain tumor glioblastoma multiforme [35]. Furthermore, F98 glioma cells are considered highly radioresistant [35,36]. This cell line is well established in our laboratory for both in vitro and in vivo studies. Thus, we can potentially follow up this in vitro experiment with an in vivo study. The cells were cultivated in growth medium containing DMEM (31966-21, Gibco), 10% fetal bovine serum and 1% penicillin/streptomycin mixture and harvested after aspirating the growth medium and incubating for approximately 20 min in a calcium- and magnesium-free medium in a standard incubator.

Exponentially growing F98 cells were split into seven groups. The cells in these groups were irradiated according to the same schedules used in the in vitro study. The samples in Group 1 and Group 4 were submitted to 5 × 4 Gy fractions of broad beam irradiation (BB), administered on five subsequent days. The samples in Group 2 and Group 5 received one single fraction of MRT only, administered on the last day of the irradiation schedule in Group 2 and on the first day of the irradiation schedule in Group 5. The samples in Group 3 and Group 6 received BB of 4 Gy daily for four days, in Group 3, the last irradiation fraction was delivered as MRT SIB and in Group 6, the first irradiation fraction was delivered as MRT SIB. The samples in Group 7 served as non-irradiated controls.

### 4.6. Analysis

Clonogenic assay: Into T25 flasks, 200 F98 glioma cells were seeded 24 h prior to the first day of irradiation, taking care to achieve a homogenous distribution of single cells across the bottom of each flask. Thus, each viable cell could generate its own colony.

The clonogenic assay samples were submitted to the same irradiation schedule as described above. Six days after the last irradiation, the colonies were terminated, adding a 10% buffered formaldehyde solution and stained with 1% Cresyl violet. Each colony with a size of 50 cells or more was counted, assuming that each colony had arisen from one single glioma cell. The data were analysed using the unpaired *t*-test (GraphPad Prism software).

Histology and immunohistochemistry: The formalin-fixed brains were sectioned 5.0 µm thick, mounted on microscope slides (SuperFrost^®^ Plus, R. Langenbrinck, Germany) for Nissl staining or with gamma H2AX antibody as described previously [37]. Briefly, the tissue sections were deparaffinised and rehydrated by passing them through a series of alcohol and xylene washes, followed by vapour-based heat epitope retrieval in a citrate solution at pH6 (Target retrieval solution, Dako, Germany) at a temperature of 95 °C for 40 min. The tissue sections were then blocked with 100 μL of 1× PBS, 5% goat serum, and 0.3% Triton X-100 buffer for 60 min at room temperature, followed by incubation with gamma H2AX (Abcam 22551, Cambridge, UK) as the primary antibody at a dilution of 1:100 for 1 h at room temperature. Finally, the tissue sections were incubated with Alexa Fluor 488 at a dilution of 1:200 (Thermo Fisher Scientific) as the secondary antibody and DAPI for 1 h at room temperature in the dark. After rinsing thoroughly with PBS, the slides were cover-slipped with Dako Fluorescent Mounting Medium (Dako North Amerika Inc., Carpinteria, CA, USA) and stored in the fridge.

This immunostaining utilises antibodies against the histone 139 which is only accessible after DNA-double-strand-breaks like those subsequent to irradiation. We have shown previously that the gamma H2AX antibody with a DAPI nuclear counterstain can be used as a reliable parameter in the assessment of DNA damage after MRT [37]. Microphotographs were obtained using a fluorescence microscope (Olympus BX61) with an attached camera (Olympus DP80) and computer link. For the immunofluorescence of the gamma H2AX stain, the excitation wavelength was 544 nm with an emission at 488 nm.

## 5. Conclusions

The results of our study could be a first suggestion that, if conventional broad beam radiotherapy is combined with an MRT SIB the latter, and administered early rather than late in the course of radiotherapy, this can elicit more efficient results than broad beam radiotherapy alone. Further data are required to decide on the optimal dose concept and timing of the MRT boost.

## Data Availability

Data as reported in this study, further inquiries can be made to the primary investigator (E.S.).
